# Comparison of the effect of omega-3 supplements and fresh fish on lipid profile: a randomized, open-labeled trial

**DOI:** 10.1038/s41387-017-0007-8

**Published:** 2017-12-19

**Authors:** Mohammad Javad Zibaeenezhad, Maryam Ghavipisheh, Armin Attar, Amir Aslani

**Affiliations:** 1 Cardiovascular Research Center, Shiraz University of Medical Sciences, Shiraz, Iran; 2 Student Research Committee, Shiraz University of Medical Sciences, Shiraz, Iran; 3 Cardiovascular Research Center, TAHA clinical trial group, Shiraz University of Medical Sciences, Shiraz, Iran

## Abstract

**Background:**

Dietary fish is a rich source of Omega-3 poly-unsaturated fatty acids (PUFAs). These compounds may have protective effect against cardiovascular events possibly by modifying lipid profiles. Consequently, fish oil supplements are produced commercially to complement low fish intake. It is not clear if both interventions have similar effects. The aim of this trial was to compare the anti-hyperlipidemic effect of omega3 fatty acid supplements with fresh fish.

**Method:**

A total of 106 patients with hyperlipidemia were randomized. One group received 2 g/day of omega-3 capsules for a period of 8 weeks and the other group received a mean of 250 g trout fish twice weekly (for dinner and lunch) for the same time period. The effects of these diets on the lipid profile after the intervention were compared between the two groups.

**Results:**

Data from 48 patients in fish oil group and 47 patients from fish group was used for final analysis. In both groups, total cholesterol, non-HDL cholesterol, triglyceride (TG) levels, and Castelli I index (total cholesterol/HDL ratio) were reduced significantly following the treatment; however, dietary-fish intake had a more pronounced effect (−85.08 ± 74.82 vs. −30.75 ± 89.00, *P* < 0.001; 75.06 ± 35.43 vs. −16.93 ± 40.21, *P* < 0.001; −66.55 ± 30.79 vs. −12.7 ± 35.48, *P* = 0.003; and −0.77 ± 1.39 vs. −3.02 ± 1.85, *P* < 0.001; respectively). HDL level was increased in both groups with a higher effect in dietary fish group (4.47 ± 7.83 vs. 8.51 ± 8.79, *P* = 0.022). Atherogenic (Log [TG/HDL ratio]) and Castelli II (LDL/HDL ratio) indices did not change in fish oil group while were reduced significantly by fresh fish consumption (−0.04 ± 0.27 vs. −0.26 ± 0.17, *P* < 0.001; and 0.15 ± 0.7 vs. -1.32 ± 1.15, *P* < 0.001, respectively). LDL level was increased in the supplementation group, while it was significantly reduced in the dietary-fish group (+18.7 ± 24.97 vs. −22.75 ± 27.28, *P* < 0.001).

**Conclusion:**

Consumption of fresh fish seems to be superior in positively modifying the lipid profiles which may have important translations in the occurrence of cardiovascular events.

## Introduction

Cardiovascular disease (CVD) is the major cause of death worldwide with an increasing trend in developing countries. The general risk factors associated with the diseases are known to be hypertension, smoking, and hyperlipidemia^1, 2^. Worldwide, high cholesterol levels cause some 56% of ischemic heart disease and 18% of strokes, amounting to 4.4 million deaths annually. It has been shown that 88 mg/dl increase in fasting circulating triacylglycerol (TG) levels elevates the risk of developing CVD by 14 and 37%, in males and females, respectively^3–5^. A 1-mg/dl increase in the low-density lipoprotein (LDL) level associates with a 2–3% increase in risk for CVDs, and elevations earlier in life may be associated with higher increases in risk. High-density lipoprotein (HDL) cholesterol independently predicts CVDs as well. Every 1-mg/dl decrease in HDL-cholesterol causes a 3–4% increase in the risk^3, 4^. Furthermore, other indices such as cholesterol ratio or Castelli I index (ratio of total cholesterol to HDL-cholesterol), Castelli II index (LDL/HDL), and atherogenic index (log of Triglyceride to HDL-cholesterol ratio) may predict CVD risk better than LDL alone^6^. So, modifying the lipid profile has become one of the most important goals in preventive cardiology. This can be achieved via medical therapy or adding beneficial dietary sources to daily regimen. Among the promising dietary sources those which are enriched in omega-3 poly-unsaturated fatty acids (PUFA) are shown to have considerable importance.

Many epidemiological studies have suggested the beneficial effects of omega-3 PUFAs on cardiovascular health^7–10^. Dietary fish is a rich source of Omega-3 PUFAs. Omega-3 PUFAs come in several forms but eicosapentaenoic acid (EPA) and docosahexaenoic acid (DHA) have been most widely investigated with regard to their cardiovascular benefits. In this regard, the American Heart Association (AHA) has endorsed their nutritional value for the secondary prevention of cardiovascular events in patients with documented coronary heart disease (CHD)^11^. They recommend that patients with CHD should consume a total of 1 g per day of DHA and EPA, preferably from oily fish. However, the fish oil omega-3 supplements in the form of capsules or liquid have also been considered as an acceptable alternative^1^. In addition, supplementation of 4 g per day omega-3 fatty acids has been considered as an approved treatment for patients with very high triglyceride levels by the Food and Drug Administration (FDA). FDA has also approved an omega-3 supplement called Lovaze which contains 456 mg EPA and 375 mg DHA for hyperlipidemia patients. Based on Omega-3 PUFAs favorable effects on lipid profiles, AHA has recommended 2–4 g daily intake of DHA and EPA for patients with high triglyceride levels^11^.

Although there are many reports on the positive effects of EPA and DHA on cardiovascular events, there are very few studies addressing which of omega-3 sources can serve this purpose the best. Dietary oily fish is a rich source of Omega-3 PUFAs. Consequently, fish oil supplements are produced commercially to complement low fish intake regimens. However, it is not clear if both sources have similar effects. In this study, we aimed to address this question by comparing the effect of dietary fish with omega-3 supplements on the lipid profile of patients suffering from hyperlipidemia.

## Methods and patients

### Trial design

This open-labeled, randomized trial was performed on 106 hyperlipidemic patients referring to Shiraz Healthy Heart House during a period of 6 months from 6 April 2015 to 12 October 2015. Medical Ethic Committee of Shiraz University of Medical Sciences approved the protocol of the study, and a signed written informed consent was obtained from each participant. The trial was registered at Iranian registry of clinical trials (www.IRCT.IR) under the registration number of IRCT201411141525N5

### Study population

Patients with hyperlipidemia who referred to Shiraz Heart House were recruited. All participants were enrolled in the trial after a complete physical examination and medical history investigation in the hospital. Adults with LDL levels = 150–190 mg/dl or cholesterol levels >250 mg/dl or TG levels = 200–500 mg/dl were included in the study. The following patients were excluded from the study: 1—pregnant patients; 2—patients with systemic diseases; 3—patients who couldn’t tolerate the supplement or the fish intake; 4—patients who needed to be treated with statins for primary or secondary prevention; and 5—all patients already taking lipid lowering drugs.

### Randomization

Randomization was done using a computer-based random digit generator based on the registration number of the patients (on the order of referral). The allocation was done in a 1:1 ratio. Apart from the project coordinator and the patients, the staff involved in clinical center, and members collecting and analyzing data were blinded to the intervention allocation.

### Interventions and outcomes

106 eligible people were randomly assigned to the dietary-fish or omega-3 supplement groups, each consisting of 53 patients. Patients of the dietary-fish group were asked to consume 250 g farmed trout fish (which contained 1.4 g omega-3 (280 mg EPA and 160 mg DHA) per 100 g) two times a week for dinner and lunch for 2 months (Equal to consumption of 14 g of omega-3 [2.8 g EPA and 1.6 g DHA] per week) while they were advised not to consume fish oil supplement during the intervention period. The patients of the supplement group received 2 g of omega-3 supplement (21st century, USA; containing 180 mg EPA and 120 mg DHA in each soft-gel) every day (Equal to consumption of 14 g of omega-3 [2.5 g EPA and 1.7 g DHA] per week) for 2 months. All participants were instructed to maintain their habitual dietary style and physical activities during the trial. All the patients were regularly checked by phone calls to see if they are still adherent to the intervention or not?

The primary outcomes were lipid profiles changes including total cholesterol, triglyceride, HDL, LDL, and total cholesterol/HDL ratio at the beginning and 2 months after starting the intervention (Just after intervention was finished). Collection and analyses of all clinical and laboratory data were performed by the study personnel blinded for group assignment.

### Measurements

The serum triglyceride was measured by GPO-PAP method providing a normal upper limit of 200 mg/dl (2.3 mmol/l). The total cholesterol was also checked by CHOD/PAP technique, which provided an upper limit normal value or 220 mg/dl (5.6 mmol/l). The HDL cholesterol was measured by dextran magnesium sulfate. LDL was measured directly using Solubilization LDL-C assay (SOL; Kyowa Medex)^12^. Some lipid profile indices were measured by following formulas:

$$\begin{array}{l} {\rm Castelli}\;{\rm I}\; ({\rm Cholesterol}\; {\rm ratio})\; {\rm Index} = {\rm Total}\; {\rm cholesterol} / {\rm HDL} \\ {\rm Castelli}\; {\rm II}\; {\rm Index} = {\rm LDL} /{\rm HDL}\\ {\rm Atherogenic}\; {\rm Index}= {\rm Log}\; ({\rm TG}/{\rm HDL}) \\ {\rm Non-HDL}\; {\rm cholesterol} = {\rm Total}\; {\rm Cholesterol} - {\rm HDL}\end{array}$$

### Statistical analysis

In order to have 90% power to detect significant differences between changes of lipid profiles, 40 patients were needed to be required in each study group (*P* < 0.05, two-sided). To compensate for possible noncompliant patients and those who would possibly leave the study, we set the total sample size at 100. All principal analyses were performed with the intention to treat population, which consisted of all the patients who underwent randomization, regardless of the treatment received. The patients who were lost to follow-up and in whom no known event had occurred were not included in the denominator for calculation of binary end points. All statistical analyses were performed using the statistical Package for Social Sciences version 17.0 (SPSS Inc., Chicago, IL, USA). Baseline characteristics were analyzed by independent *t*-test or Chi-square tests. The 95% confidence intervals for the means of data were calculated, and the significance of the differences between results within groups was assessed using paired t-tests. A two-sided *P* value < 0.05 was considered statistically significantly. We also used a covariance analysis in order to minimize the effect of the baseline differences whenever needed.

## Results

**Fig. 1:**
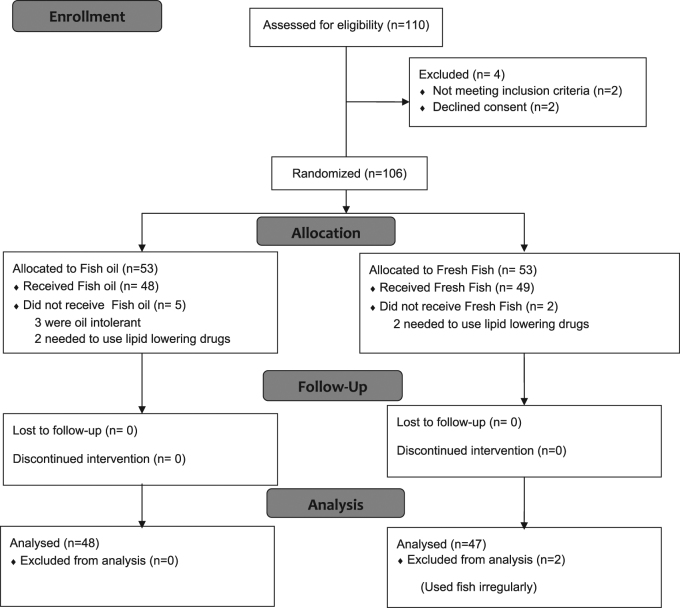
**CONSORT 2010 flow diagram for this randomized, double-blinded, clinical trial of effects of fish oil in comparison of fresh fish on lipid profiles in hyperlipidemic patients**

Of the 53 patients enrolled in the supplement group, 5 were excluded due to gastrointestinal oil intolerance, and a new need to use lipid-lowering drugs. Of 53 patients in the dietary-fish group, 4 were excluded from the study due to noncompliance with fish intake or a new need to use lipid-lowering drugs and two others were excluded from analysis because they consumed fish irregularly. Consort 2010 flow diagram is shown in Fig. 1.

There were no significant differences in terms of the patients’ gender between the two groups (*P* = 0.5). As shown in Table 1, the patients’ systolic and diastolic blood pressures, body mass index (BMI), and age was not different between the two groups at the beginning of the study and before our interventions. Prior to our interventions, the levels of cholesterol, LDL, HDL, and total cholesterol/HDL ratio were significantly different between the groups (Table 1). Therefore, we used the covariance analysis in order to minimize the effect of these differences.

**Table 1:** Comparison of the mean age, BMI, SBP and DBP between the two groups at baselin

Variables	Omega-3 group (*n* = 48)	Dietary-fish group (*n* = 47)	*P*-value
Age (years)	48.7	50.73	0.257
*Sex*			0.5
Male (%)	25 (52.1%)	23 (48.9%)	
Female (%)	23 (47.9%)	24 (51.1%)	
Body mass index (Kg/m^2^)	26.24	26.57	0.901
Systolic blood pressure (mmHg)	173 ± 12	164 ± 12	0.47
Diastolic blood pressure (mmHg)	70 ± 8	71 ± 13	0.54
*Lipid profiles*			
Total cholesterol (mg/dl)	230.20 ± 43.11	251.65 ± 47.87	0.023
TG (mg/dl)	270.02 ± 64.81	294.71 ± 109.56	0.181
LDL (mg/dl)	114.72 ± 25.15	133.61 ± 34.57	0.003
HDL (mg/dl)	42.34 ± 7.23	36.14 ± 9.27	0.001

*HDL* high density lipoprotein, *LDL* low density lipoprotein, *TG* triglyceride

Our results indicated that treatment by both omega-3 supplements and fresh fish caused a significant decrease in total cholesterol, non-HDL cholesterol, TG levels, and total cholesterol/HDL ratio but this reduction was more prominent in the fresh fish group. Blood LDL was another factor which was measured between the two groups. The fish oil supplement not only had no beneficial effect on the LDL level but also worsened the situation by significantly increasing the LDL level (18.95 ± 30.46%). However, in the dietary-fish group the LDL level was significantly decreased (−15.25 ± 17.97%, *P* < 0.001). HDL level was increased in both groups; again, dietary-fish was more effective in raising the HDL level (11.51 ± 17.93% vs. 27.39 ± 26.76%; *P* = 0.001). Both Atherogenic and Castelli II indices showed no significant change with fish oil supplementation but depicted significant decreases with fresh fish consumption. All the comparisons are shown in Table 2.

**Table 2:** Mean values (Mean ± SD) for lipids levels in intervention and control groups before and after the study

Lipid Parameters	Omega-3 group (*n* = 48)				Dietary-fish group (*n* = 47)				Treatment difference	95% confidence interval	*P* value
	Before	After	unit Change	*P* value	Before	After	unit change	*P* value			
Triglyceride (mg/dl)	270.02 ± 64.81	239.27 ± 91.46	−30.75	0.021	294.71 ± 109.56	209.63 ± 104.73	−85.08	0.001	−54.33	−87.45 to −21.20	<0.003
Total cholesterol (mg/dl)	230.20 ± 43.11	217.5 ± 42.92	−12.7	0.017	251.65 ± 47.87	185.1 ± 38.63	−66.55	0.001	−53.84	−67.23 to −40.45	<0.001
Low density lipoproteins (LDL) (mg/dl)	114.72 ± 25.15	133.43 ± 34.42	18.7	0.001	133.61 ± 34.57	110.85 ± 28.73	−22.75	0.001	141.46	−52.01 to −30.91	<0.001
High density lipoproteins (HDL) (mg/dl)	42.34 ± 7.23	46.33 ± 8.94	4.47	<0.001	36.14 ± 9.27	44.65 ± 8.82	8.51	<0.001	4.03	0.58 to 7.48	0.022
Non-HDL cholesterol (mg/dl)	186.09 ± 43.16	169.15 ± 41.74	−16.93	0.008	215.51 ± 41.26	140.45 ± 35.53	−75.06	<0.001	58.12	42.54 to 73.71	<0.001
Total cholesterol to HDL ratio (cholesterol ratio, Castelli I Index)	5.42 ± 1.28	4.78 ± 1.09	−0.77	<0.001	7.26 ± 1.81	4.24 ± 0.91	13.02	<0.001	−2.24	−2.93 to −1.56	<0.001
LDL to HDL ratio (Castelli II index)	2.70 ± 0.63	2.86 ± 0.78	0.15	0.162	3.84 ± 1.15	2.52 ± 0.62	−1.32	<0.001	−1.47	−1.87 to −1.07	<0.001
Log (triglyceride to HDL ratio) (Atherogenic index)	0.71 ± 0.27	0.67 ± 0.20	−0.04	0.27	0.90 ± 0.17	0.63 ± 0.22	−0.26	<0.001	−0.22	−0.31 to −0.13	<0.001

## Discussion

Here, by comparing the effect of omega-3 supplements and dietary fish on the blood lipid profile and indices of patients with hyperlipidemia, we have shown that dietary-fish intake had a significantly more pronounced effect on lowering the total cholesterol, non-HDL cholesterol, LDL, and TG levels; and indices of Castelli I (total cholesterol/HDL ratio), Castelli II (LDL/HDL ratio), and Atherogenic index (log [TG/HDL ratio]). In the case of HDL, fresh fish more effectively raised its level.

Populations whose diets are based on regular fish intake (like Greenland Eskimos) have been noted to have a lower incidence of CHD^13^. This effect had been contributed to the presence of high amounts of omega-3 PUFAs in the fish^14–16^. Omega-3 PUFAs come in several forms but EPA and DHA have been most widely investigated with regard to their cardiovascular benefits. DHA and EPA are suggested to enrich the biological membrane phospholipids^17^; hence, they improve the arterial and endothelial function^18^. They are also reported to reduce the platelet aggregation^19^ and blood pressure^20, 21^. Omega-3 PUFAs may also reduce insulin resistance and suppress the pro-inflammatory responses triggered by cytokines^22^.

Several studies have addressed the question that how omega-3 supplementation would affect lipid profile. However, findings about this issue are highly controversial. In a study by Wei and colleagues, it was shown that LDL levels were elevated by 5% after DHA consumption, while those consuming EPA had a non-significant reduction in serum LDL of 1%^23^. In a systematic review, Eslick and colleagues showed that 3.25 g/day consumption of fish oil (1.9 g of EPA and 1.35 g of DHA) reduced the serum TG by 14%, while the LDL-c was not significantly increased^24^. Similar results were obtained in systematic reviews by Balk and Mori, both of which addressed the studies in which individuals consumed either fish, algal EPA or algal DHA oils^25, 26^. Leslie and coworkers reviewed the beneficial effect of omega-3 on circulating TG levels in normolipidemic to borderline hyperlipidemic healthy individuals. Their results indicated 9-26% reduction in circulating TG level where more than 4 g/day of omega-3 was consumed from either marine or EPA/DHA-enriched food sources, while a 4–51% reduction was found where 1–5 g/day of EPA and/or DHA was consumed through supplements^27^. In a randomized clinical trial, Yu Qin and colleagues found that 4 g of fish oil supplement consumption for 3 months effectively decreased the serum total cholesterol, triglyceride, apolipoprotein B and glucose concentrations in nonalcoholic fatty liver disease patients with hyperlipidemia^28^. In general, it is believed that fish oil consumption may decrease the TG level by 25–30%^19^. These findings are in agreement with ours. We saw nine percent TG reduction with fish oil consumption. The observed variable effects may arise from different dosages and sources used as the intervention in those studies. We know that different dosages of omega-3 supplements have different effects. For example, for patients with diagnosed ischemic heart disease, the AHA recommends about 1 g of EPA + DHA per day. For patients with elevated serum triglyceride levels, a higher dose of EPA + DHA, 2–4 g/day, is recommended. However, some experts believe that this recommendation should be revised to 3–4 g/day^11, 29^. Here we used a dosage of 2 g per day as our intervention. However, dietary fresh fish resulted in 29% TG reduction which is similar to effects of high dose fish oil supplements in previous studies. It should also be noticed that Omega-3 supplements also do contain DPA and increasing DPA may also be related to TG lowering effect of these supplements^12^.

Since many of food and supplement-based studies have utilized different sources and doses of omega-3 provided as fish oil supplements or dietary fish, it is not clear which form is bioactive in affecting the serum lipid levels^27^. Only few studies have taken into consideration the point that using an omega-3 supplement may not have the same effects as using fresh fish. In a randomized controlled trial study, it was shown that in patients who received omega-3 PUFAs after a myocardial infarction, the mortality rate was reduced by 29% with either using the oily fish (about 300 g per week) and fish-oil capsules (about 900 mg of DHA + EPA per day)^30^; although, no direct comparison was done between dietary fish and oil supplements. Here, we have noticed that fresh fish is far better than omega-3 supplements in modifying lipid profiles. This may be due to the fact that several important nutrients like selenium, vitamin D, and naturally occurring antioxidants are only found in oily fish and fish-oil supplements lack them. The dietary-fish can have another cardiovascular beneficial effect by consistently lowering the C-reactive protein levels, the effect which is absent from fish-oil supplements^25, 31, 32^. On the other hand, selenium has antithrombotic properties, reduces lipid peroxidation, myocardial infarct size, and ischemia-induced ventricular arrhythmias, and improves recovery from ischemia or reperfusion injury^33^. Moreover, the powerful antioxidant properties of selenium as well as its ability to attenuate the adverse effects of methyl mercury (which is present in fish in varying concentrations) can provide additional cardiovascular benefits^33, 34^. Finally, using fish in the regular diet means replacing a previous dietary component (such as red meat) with fish. Consequently, the probable undesirable effects of previous dietary contents are reduced, an effect that cannot be achieved by using fish oil supplement.

Our study had some limitations. In this study, we used the farmed trout fish which belongs to salmon family, as the source of dietary fish. This breed had a 1.4 g omega-3 per 100 g fish^35^. However, the routine dietary fish in our region (south of Iran) and many other areas of world do not contain this amount of omega-3 in their tissue^36^. Another important limitation of our study was that in spite of the fact that we performed a classic randomization, we found a significant difference in the baseline lipid profile of patients in the two groups. Consequently, we performed a multivariate analysis to minimize its effect. In addition, although we tried our best to supply equal amounts of omega-3 (14 g per week) to the both study groups, it was not possible to supply an exactly equal amount of EPA and DHA to both groups (2.8 g EPA and 1.6 g DHA; 2.5 g EPA and 1.7 g DHA per week for fresh fish and fish oil supplement groups respectively). That happened because EPA and DHA constituted different percentage of omega-3 acids in the fish oil and fresh fish. However, those amounts were very close and not statistically different. Another limitation was that, like other dietary studies, economic and social differences might have produced differences in lifestyle, activity level and baseline diet which made it difficult to distinguish their effects we couldn’t exclude. So in order to have more conclusive results, the enrollment of a larger population and with more diverse ethnicity background is demanded. We should also take another important note into consideration. Increasing the fish servings in a week may mean omitting or replacing other unhealthy diets such as fast foods or even diets with lower nutritional benefits such as red meats. This does not happen with adding fish oil supplement to daily diet. This effect could not be neutralized in this study as the study question demanded the current design. However, it should be noticed that the results are still highly viable. In fact, fish oil supplement cannot replace dietary fish whether the beneficial effect is driven by other fresh fish contents or by modifying the weekly pattern of nutrition. Finally, it should be noticed that we did not measure EPA, DHA or DPA levels before and after the intervention.

It can be concluded that fresh fish consumption can improve lipid profiles better than omega-3 supplementation, and using oil fish is not a substitute for fresh fish consumption. Based on our observations, consumption of fresh fish seems to be superior in lowering the total cholesterol, LDL, and TG levels as well as increasing HDL level. These effects may translate into a reduction in the risk of CVD. Future comparative large scale trials are needed to see whether these biochemical differences can be translated into clinical outcomes.
